# Very Early Discharge After Primary Percutaneous Coronary Intervention (PCI) in Low-Risk ST-Segment Elevation Myocardial Infarction (STEMI) Patients: Safety and Feasibility From a Prospective Randomized Study

**DOI:** 10.7759/cureus.113670

**Published:** 2026-07-30

**Authors:** Abdelkarim Ait Yahya, Wissame Dahmane, Mohamed Ztati, Imane Ait Lahcen, Mustapha El Hattaoui

**Affiliations:** 1 Cardiology, Mohammed VI University Hospital Center, Faculty of Medicine and Pharmacy of Marrakech, Cadi Ayyad University, Marrakech, MAR

**Keywords:** coronary intervention, early discharge, low-risk patients, mace, primary pci, stemi

## Abstract

Background: Contemporary management of ST-segment elevation myocardial infarction (STEMI) has markedly evolved with the widespread adoption of primary percutaneous coronary intervention (PCI), radial-access strategies, and optimized antithrombotic therapy. In clinically stable patients, serious post-procedural complications have become increasingly uncommon beyond the first 24 hours, raising questions regarding the necessity of prolonged hospitalization after uncomplicated STEMI. The issue becomes even more relevant in resource-limited healthcare systems, where coronary care unit capacity often remains critically constrained. Although observational studies have suggested that selected low-risk patients may safely undergo very early discharge, prospective randomized data remain limited, particularly in low- and middle-income countries.

Objective: To assess the safety and feasibility of very early discharge (≤48 hours) after successful primary percutaneous coronary intervention (PCI) in selected low-risk STEMI patients.

Methods: We conducted a prospective, randomized, open-label, single-center study between June 2024 and June 2025 at Mohammed VI University Hospital Center, Marrakech, Morocco. During the study period, 544 consecutive patients were admitted for STEMI. Among them, 179 fulfilled predefined low-risk criteria, including preserved left ventricular ejection fraction (>50%), successful radial-access PCI with final thrombolysis in myocardial infarction (TIMI) 3 flow, Killip class I presentation, limited coronary artery disease burden, and absence of major in-hospital complications during the first 24 hours. Eligible patients were randomized in a 1:1 ratio to either very early discharge (≤48 hours) or routine discharge (>48 hours). The primary endpoint was major adverse cardiovascular events (MACE) at 3 months, defined as all-cause mortality, recurrent myocardial infarction, hospitalization for heart failure, or definite/probable stent thrombosis.

Results: A total of 82 patients were assigned to the very early discharge group and 97 to routine discharge. Median hospital stay in the early discharge group was 25.8 hours. Baseline clinical and angiographic characteristics were comparable between groups, with preserved left ventricular systolic function and successful radial-access PCI achieved in all randomized patients. At the three-month follow-up, MACE occurred in 2 patients (2.4%) in the early discharge group and in 2 patients (2.1%) in the routine discharge group, with no significant difference between discharge strategies (p = 1.00). In the early discharge group, one patient (1.2%) died, and one patient (1.2%) required hospitalization for heart failure. In the routine discharge group, one patient (1.0%) experienced recurrent myocardial infarction and one patient (1.0%) required hospitalization for heart failure. No cases of stent thrombosis occurred during follow-up.

Conclusion: In this prospective randomized study involving selected low-risk STEMI patients, very early discharge (≤48 hours) after successful primary PCI was not associated with an apparent increase in adverse cardiovascular events during the three-month follow-up. Although the modest sample size and short follow-up preclude definitive conclusions regarding long-term safety, these findings support the feasibility of an individualized very early discharge strategy in carefully selected patients, particularly in resource-constrained healthcare settings.

## Introduction

ST-segment elevation myocardial infarction (STEMI) remains a major cause of cardiovascular morbidity and mortality worldwide despite substantial progress in reperfusion therapy and contemporary pharmacological management [1]. The widespread adoption of primary percutaneous coronary intervention (PCI), radial-access strategies, potent antithrombotic therapy, and early secondary prevention has considerably improved short- and long-term outcomes following STEMI [2]. As a result, successful reperfusion and rapid clinical stabilization are now frequently achieved in uncomplicated cases.

Historically, hospitalization for at least 48-72 hours after STEMI was considered necessary to monitor for arrhythmic, ischemic, or mechanical complications [3]. Yet contemporary evidence suggests that most serious adverse events following successful primary PCI occur during the first 24 hours, particularly in hemodynamically stable patients with preserved ventricular function [4]. Once this early high-risk period has passed, the incremental clinical benefit of prolonged in-hospital monitoring appears increasingly limited in carefully selected low-risk patients.

This evolution has important organizational and economic implications. Prolonged hospitalization after uncomplicated STEMI continues to contribute substantially to healthcare costs, coronary care unit occupancy, and resource utilization, especially in high-volume tertiary centers [5]. The issue becomes even more relevant in low- and middle-income healthcare systems, where limitations in intensive cardiac care capacity may directly affect access to acute cardiovascular management.

Several observational studies and randomized data have suggested that selected low-risk STEMI patients may safely undergo early discharge following successful primary PCI [4,6-8]. In this context, low-risk STEMI generally refers to clinically stable patients with successful reperfusion, preserved left ventricular systolic function, no significant in-hospital complications, and a low predicted risk of short-term adverse cardiovascular events.

Risk stratification tools such as the Zwolle Risk Score have been developed to identify patients at low risk for short-term adverse events after STEMI [9]. Current European guidelines also support consideration of early discharge in carefully selected, clinically stable STEMI patients after successful primary PCI when appropriate risk stratification and post-discharge follow-up are ensured [1]. More recently, contemporary pathway-based studies incorporating structured follow-up strategies have demonstrated the feasibility of discharge within 24-48 hours in carefully selected patients, with low rates of mortality and major adverse cardiovascular events (MACE) [4,5].

Nevertheless, prospective randomized evidence remains limited, and most available data originate from high-income healthcare systems in Europe and North America. Data regarding very early discharge strategies in resource-constrained settings remain scarce. In Morocco, restricted coronary care unit availability and financial limitations affecting both healthcare institutions and patients further amplify the impact of prolonged hospitalization. In such a context, safely reducing hospital length of stay without compromising clinical outcomes represents not only a logistical challenge but also a relevant public health objective.

We therefore conducted a prospective randomized study to evaluate the safety and feasibility of very early discharge (≤48 hours) compared with routine discharge in carefully selected low-risk STEMI patients treated with primary PCI in a tertiary care center in Morocco.

## Materials and methods

Study design and setting

We conducted a prospective, randomized, open-label, single-center study in the Department of Cardiology at Mohammed VI University Hospital Center, Marrakech, Morocco, between June 2024 and June 2025.

Study population

During the study period, 544 consecutive patients were admitted with ST-segment elevation myocardial infarction (STEMI). Among them, 179 patients met predefined low-risk criteria and were eligible for randomization. The inclusion and exclusion criteria are summarized in Table 1.

**Table 1:** Inclusion and exclusion criteria for patient selection

Inclusion criteria	Exclusion criteria
Preserved left ventricular ejection fraction (LVEF) >50%	Cardiogenic shock
Successful primary PCI with final Thrombolysis in Myocardial Infarction (TIMI) grade 3 flow	Heart failure (Killip ≥ II)
Radial arterial access	LVEF ≤50%
Single- or two-vessel coronary artery disease	Multivessel disease requiring staged revascularization
Killip class I at admission	Femoral access
Absence of in-hospital complications within the first 24 hours	Failed PCI or major in-hospital complications

Randomization and discharge strategy

Eligible patients were randomized in a 1:1 ratio using a computer-generated randomization sequence created in Microsoft Excel. At the time of enrollment, each participant was assigned a randomly selected study number corresponding to the pre-generated randomization list, which determined allocation to either the very early discharge group (≤48 hours) or the routine discharge group (>48 hours).

In the very early discharge group, hospital stay ranged between 24 and 30 hours (median: 25.8 hours), provided clinical stability was maintained. Discharge eligibility was confirmed by the treating cardiologist after verification of persistent clinical stability, absence of recurrent ischemic symptoms, hemodynamic stability, and absence of procedure-related complications.

**Figure 1:**
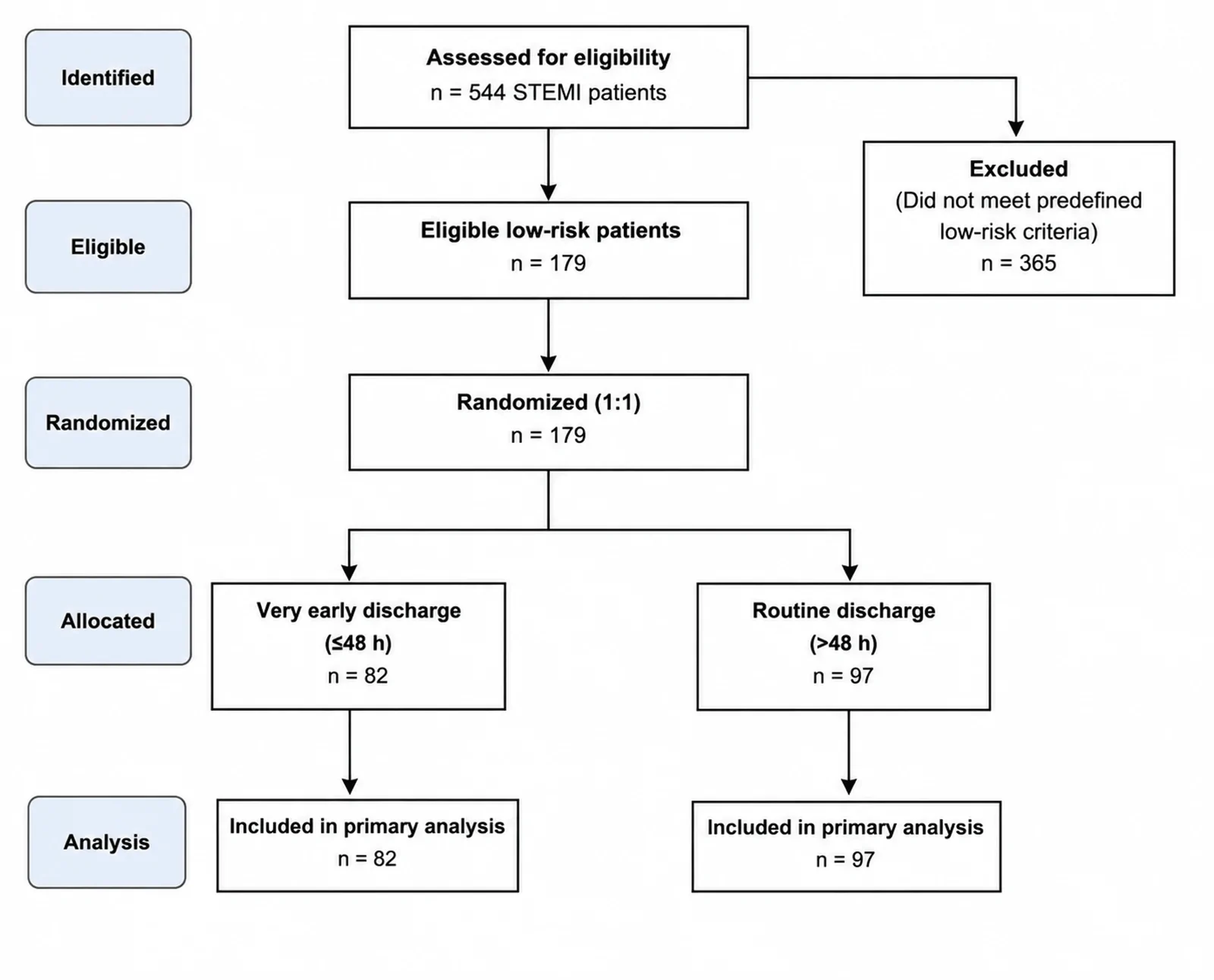
CONSORT flow diagram of the study population CONSORT flow diagram of patient selection, allocation, follow-up, and inclusion in the primary analysis after STEMI treated with primary PCI [10].

Patient selection, randomization, follow-up, and inclusion in the primary analysis are illustrated in Figure 1.

Follow-up and endpoints

All patients underwent follow-up at one week (clinical and electrocardiographic assessment), one month (clinical and echocardiographic evaluation), and at two and three months. The primary endpoint was MACE at three months, defined as a composite of all-cause mortality, recurrent myocardial infarction, hospitalization for heart failure, and definite or probable stent thrombosis.

Statistical analysis

Continuous variables were expressed as mean ± standard deviation and compared using Student’s t-test. Categorical variables were expressed as frequencies and percentages and compared using the chi-square test or Fisher’s exact test when appropriate. A two-sided p-value <0.05 was considered statistically significant. Statistical analyses were performed using IBM SPSS Statistics for Windows (IBM Corp., Armonk, NY).

Ethics statement

The study protocol was reviewed and approved by the institutional ethics committee of Mohammed VI University Hospital Center, Marrakech. All participants provided written informed consent prior to enrollment. The study was conducted in accordance with the principles of the Declaration of Helsinki.

## Results

A total of 179 patients were randomized, including 82 patients assigned to very early discharge and 97 patients to routine discharge. Only patients with complete follow-up data at three months were included in the final analysis; therefore, no loss to follow-up occurred among the analyzed study population.

Baseline characteristics

Baseline characteristics were comparable between groups. The mean age was 56 ± 2 years in the early discharge group and 55 ± 2 years in the routine discharge group (p = 0.41). Female patients accounted for 54.8% and 54.6% of the early and routine groups, respectively (χ² = 0.001, df = 1, p = 0.97). Mean left ventricular ejection fraction was 56 ± 5% in the early discharge group and 55 ± 5% in the routine group (p = 0.27). All patients underwent successful radial-access PCI. The distribution of single- and two-vessel coronary artery disease was similar between groups.

Baseline clinical and angiographic characteristics are summarized in Table 2.

**Table 2:** Baseline characteristics according to discharge strategy Continuous variables are presented as mean ± standard deviation (SD) and categorical variables as n (%). Chi-square test results are presented with corresponding degrees of freedom (df).

Variable	Early discharge (n = 82)	Routine discharge (n = 97)	p-value
Age, years	56 ± 2	55 ± 2	0.41
Female sex, n (%)	45 (54.8%)	53 (54.6%)	χ²=0.001, df=1, p=0.97
Left ventricular ejection fraction (%)	56 ± 5	55 ± 5	0.27
Radial access PCI, n (%)	82 (100%)	97 (100%)	
Single-vessel disease, n (%)	56 (68.3%)	66 (68.0%)	χ²=0.001, df=1, p=0.97
Two-vessel disease, n (%)	26 (31.7%)	31 (32.0%)	χ²=0.001, df=1, p=0.97

Clinical outcomes

At the three-month follow-up, the composite primary endpoint (MACE) occurred in two patients (2.4%) in the early discharge group, and in two patients (2.1%) in the routine discharge group (p = 1.00, Fisher’s exact test).

In the early discharge group, one patient (1.2%) died, and one patient (1.2%) was hospitalized for heart failure. In the routine discharge group, one patient (1.0%) experienced recurrent myocardial infarction and one patient (1.0%) was hospitalized for heart failure. No cases of stent thrombosis were recorded in either group.

Overall, the three-month mortality in the entire cohort was 0.55%. Clinical outcomes at the three-month follow-up are summarized in Table 3.

**Table 3:** Clinical outcomes at the three-month follow-up Values are presented as n (%). *Fisher’s exact test. MACE: major adverse cardiovascular events.

Outcome	Early discharge (n = 82)	Routine discharge (n = 97)	p-value
MACE (composite), n (%)	2 (2.4%)	2 (2.1%)	1.00
All-cause mortality, n (%)	1 (1.2%)	0 (0.0%)	0.46*
Recurrent myocardial infarction, n (%)	0 (0.0%)	1 (1.0%)	1.00*
Hospitalization for heart failure, n (%)	1 (1.2%)	1 (1.0%)	1.00
Stent thrombosis, n (%)	0 (0.0%)	0 (0.0%)	—

**Figure 2:**
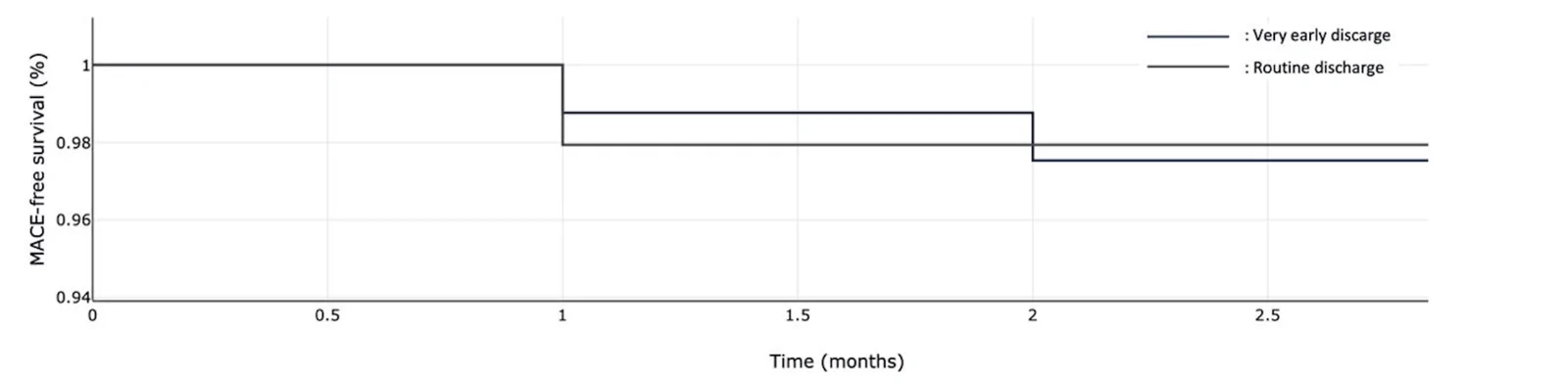
Kaplan-Meier curve for three-month MACE-free survival according to discharge strategy Comparison of the three-month MACE-free survival between very early discharge and routine discharge groups after primary PCI for STEMI.

Kaplan-Meier analysis of the three-month MACE-free survival is illustrated in Figure 2, showing no significant difference between the groups (log-rank p = 0.86).

## Discussion

In this prospective randomized study conducted in a tertiary care center in Morocco, very early discharge within 48 hours after successful primary PCI in carefully selected low-risk STEMI patients was not associated with an increased risk of adverse cardiovascular events at three months. The overall incidence of MACE remained low in both groups, with no significant differences in mortality, recurrent myocardial infarction, hospitalization for heart failure, or stent thrombosis between discharge strategies.

These findings are consistent with previous observational and randomized studies supporting the safety of early discharge after uncomplicated STEMI in selected low-risk patients [4-8]. In a prospective randomized trial involving low-risk STEMI patients treated with primary PCI, Novobilský et al. demonstrated that discharge within 48-72 hours was feasible and not associated with increased 90-day adverse cardiovascular outcomes compared with standard discharge [6]. Similarly, Rathod et al. reported very low mortality and MACE rates among patients discharged within 48 hours following successful primary PCI using a structured multidisciplinary follow-up pathway [4]. More recently, larger contemporary cohorts have confirmed the long-term safety and cost-effectiveness of this strategy in appropriately selected patients [5].

The rationale supporting very early discharge after STEMI is closely linked to the evolution of contemporary reperfusion management. Radial-access PCI, high rates of successful reperfusion, optimized antiplatelet therapy, and improved secondary prevention have substantially reduced the incidence of early post-infarction complications [2,4]. Importantly, major arrhythmic and mechanical complications requiring urgent intervention now occur predominantly during the initial 24 hours after presentation, particularly in clinically stable patients with preserved left ventricular systolic function [4]. Beyond this period, prolonged hospitalization may provide limited additional prognostic benefit in carefully selected low-risk patients.

Careful patient selection nevertheless remains essential. In our study, low-risk status was defined using strict clinical, angiographic, and echocardiographic criteria, including preserved left ventricular ejection fraction, successful radial-access PCI with final TIMI 3 flow, Killip class I presentation, limited coronary artery disease burden, and absence of early in-hospital complications. These criteria are broadly consistent with previously validated early discharge pathways and contemporary guideline recommendations [1,4,9]. The Zwolle Risk Score, initially developed to identify STEMI patients eligible for shortened hospitalization, remains one of the most widely validated risk stratification tools in this setting [9]. In addition, preserved left ventricular systolic function has consistently been associated with lower short-term complication rates and may further refine discharge decision-making [11].

Recent evidence also highlights the importance of structured post-discharge management in optimizing outcomes after acute coronary syndromes. Medication adherence following myocardial infarction remains a major determinant of long-term prognosis, and premature discontinuation of antiplatelet therapy has been associated with increased risks of recurrent ischemic events and mortality [12]. Several studies have demonstrated that improved adherence to cardiovascular therapies significantly reduces adverse cardiovascular outcomes after acute coronary syndromes [12,13]. In parallel, contemporary telemedicine and remote follow-up strategies have shown promising results in post-PCI care, with improved clinical monitoring, patient satisfaction, and optimization of secondary prevention therapies [14,15]. These findings may further support the feasibility of very early discharge strategies when combined with dedicated multidisciplinary follow-up programs and careful patient selection. In addition, early discharge after successful primary PCI has previously been shown to be safe and cost-effective in low-risk myocardial infarction patients, reinforcing the potential organizational and economic benefits of this approach [16].

The present study also highlights the potential relevance of early discharge strategies in resource-limited healthcare systems. Most published data originate from high-income countries with substantial organizational and technological resources. In contrast, our study was conducted in a setting where coronary care unit capacity and hospital resources remain constrained. Under such circumstances, safely reducing hospital stay may improve bed availability and optimize healthcare resource utilization without compromising patient safety. This consideration may be particularly important in high-volume tertiary referral centers managing large numbers of acute coronary syndromes.

Study limitations

Several limitations should nevertheless be acknowledged. First, although prospective and randomized, it was conducted at a single center with a relatively modest sample size, and no formal a priori sample size calculation was performed, which may have limited the statistical power to detect differences in infrequent clinical events. Second, the open-label design without blinding may have introduced potential bias, although the primary outcomes were objective clinical endpoints. Third, the follow-up period was limited to three months, precluding assessment of longer-term safety and clinical outcomes. In addition, a formal allocation concealment procedure was not implemented, which may have introduced a potential risk of selection bias. Finally, the trial was not prospectively registered, which should be considered when interpreting the findings. 

## Conclusions

In this prospective randomized study, very early discharge within 48 hours after successful primary PCI in carefully selected low-risk STEMI patients was not associated with an increased risk of adverse cardiovascular events at three months. These findings support the short-term safety and feasibility of an individualized early discharge strategy following uncomplicated STEMI. In resource-constrained healthcare systems, such an approach may help optimize hospital bed utilization and coronary care unit capacity without compromising short-term clinical outcomes.
